# 

**Published:** 1896-03

**Authors:** 


### CONTENTS FOR MARCH, 1896.

ORIGINAL COMMUNICATIONS. PAGE.

The Principles of Preventive Inoculation and Serum Therapeutics, with Considerations Concerning their Practical Application. By A. C. Abbott, M. D. .... 609
Laboratory Experience in the Bacteriology of Diphtheria. By Wm. G. Bissell, M. D... 626
A Pseudencephalic Monster. By Wm. L. Conklin, M. D .... 633
A Case of Rhinoscleroma Originating in the United States. By Grover Wm. Wende, M. D .... 642
Treatment of Diphtheria. By Frederic W. Bartlett, M D .... 647

PROGRESS IN MEDICAL SCIENCE.

Ophthalmology .... 650
Genito-Urinary and Syphilitic Diseases .... 653
Obstetrics, Gynecology and Pelvic Surgery .... 657

SOCIETY PROCEEDINGS.

Medical Society of the County of Erie .... 663

EDITORIAL.

The Medical Society of the State of New York .... 665
Topics of the Month .... 667

Personal .... 675
Obituary .... 676
Society Meetings .... 678
Reviews .... 679
Literary Notes .... 686
Miscellany .... 687

